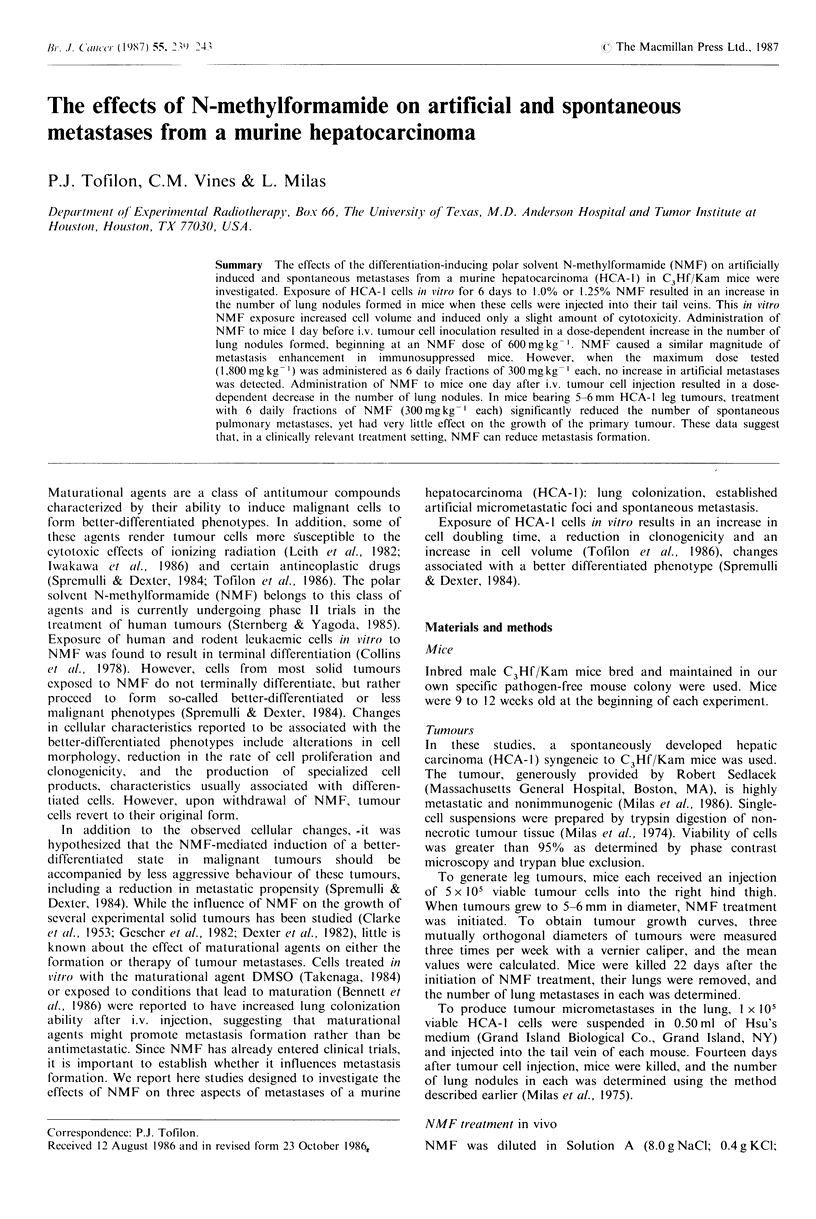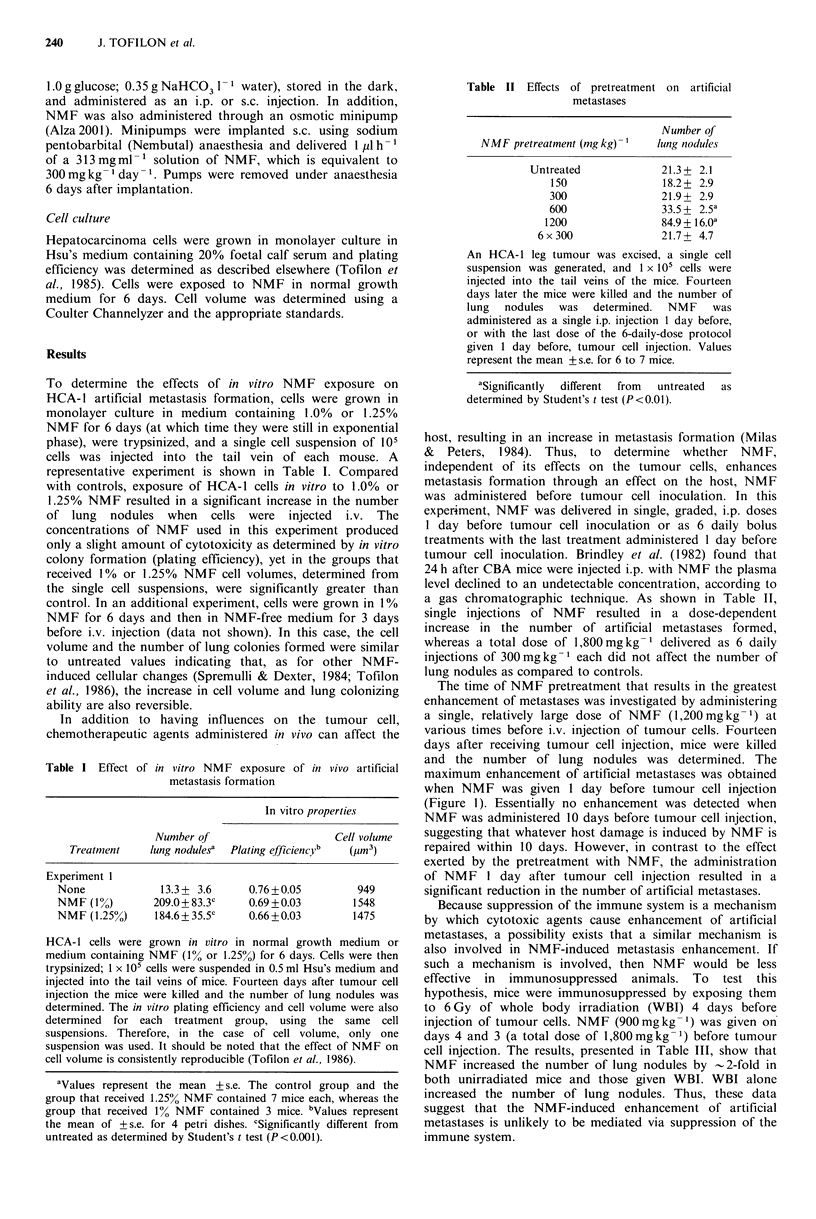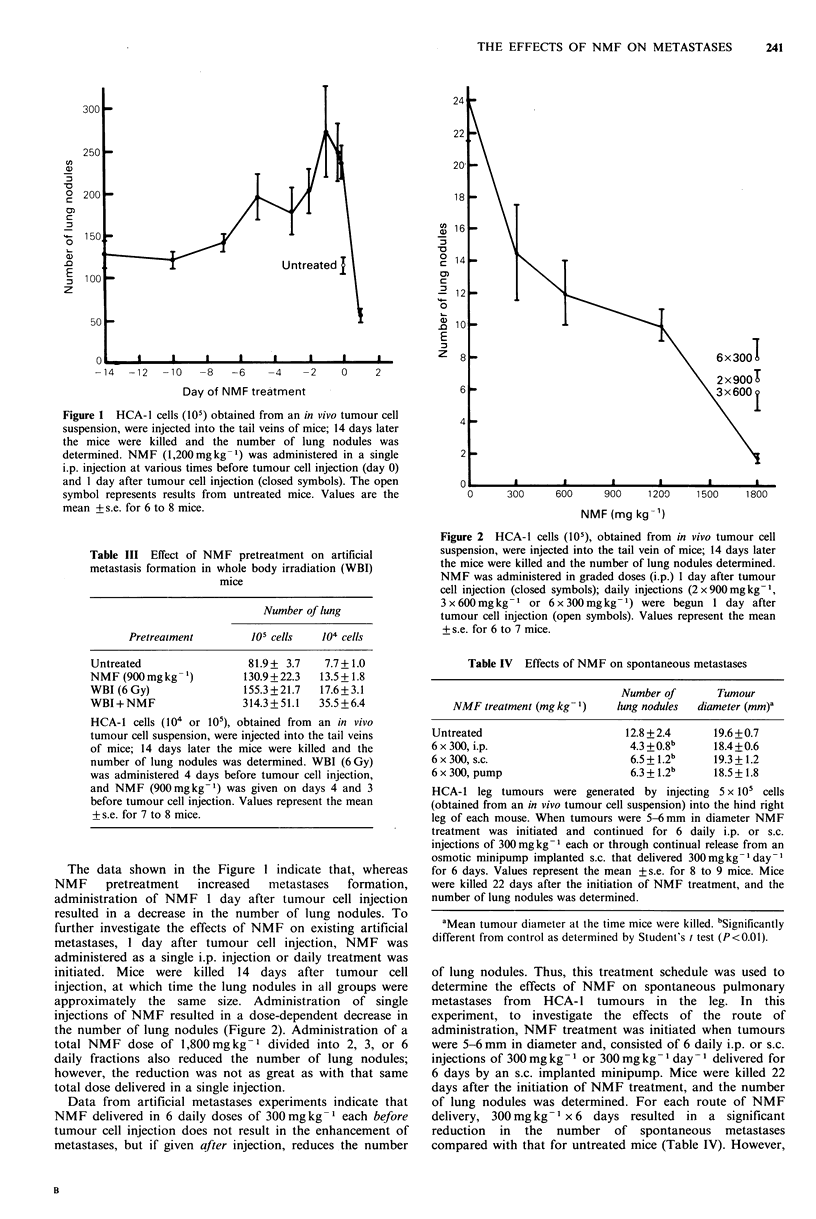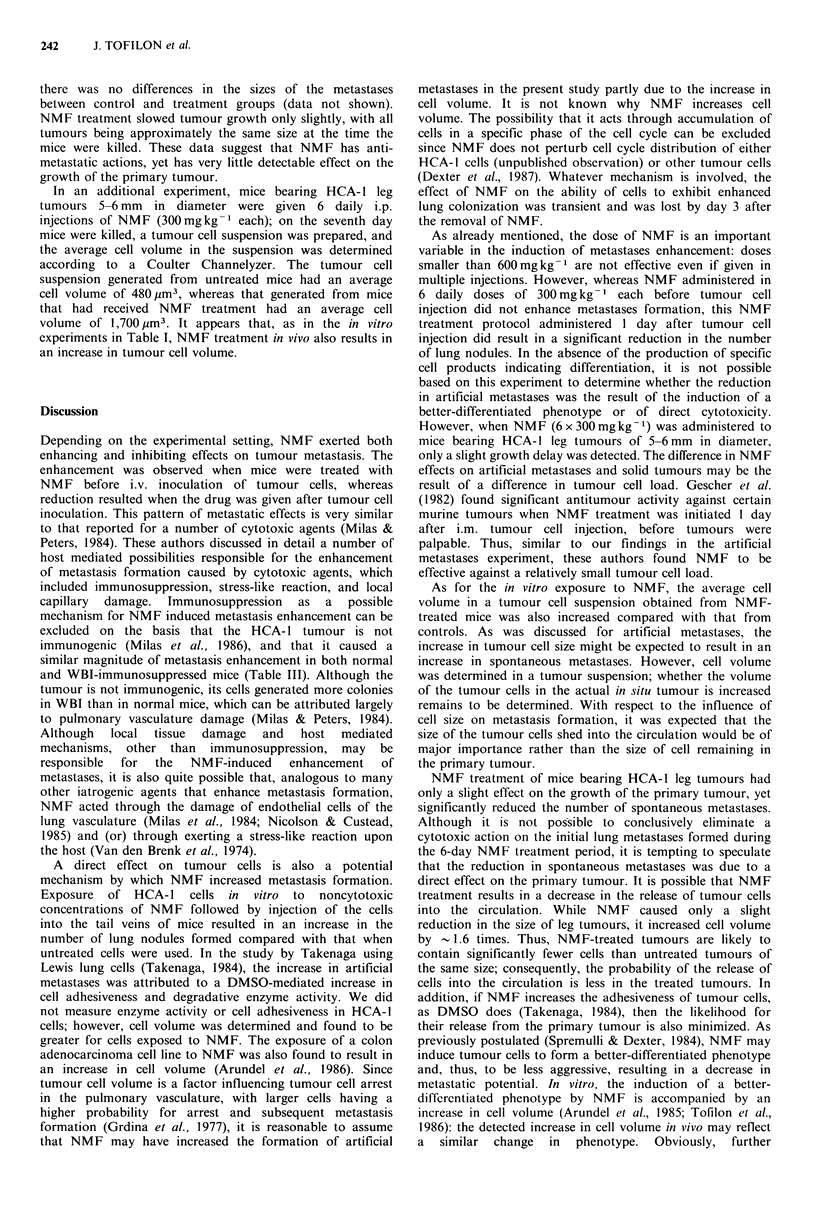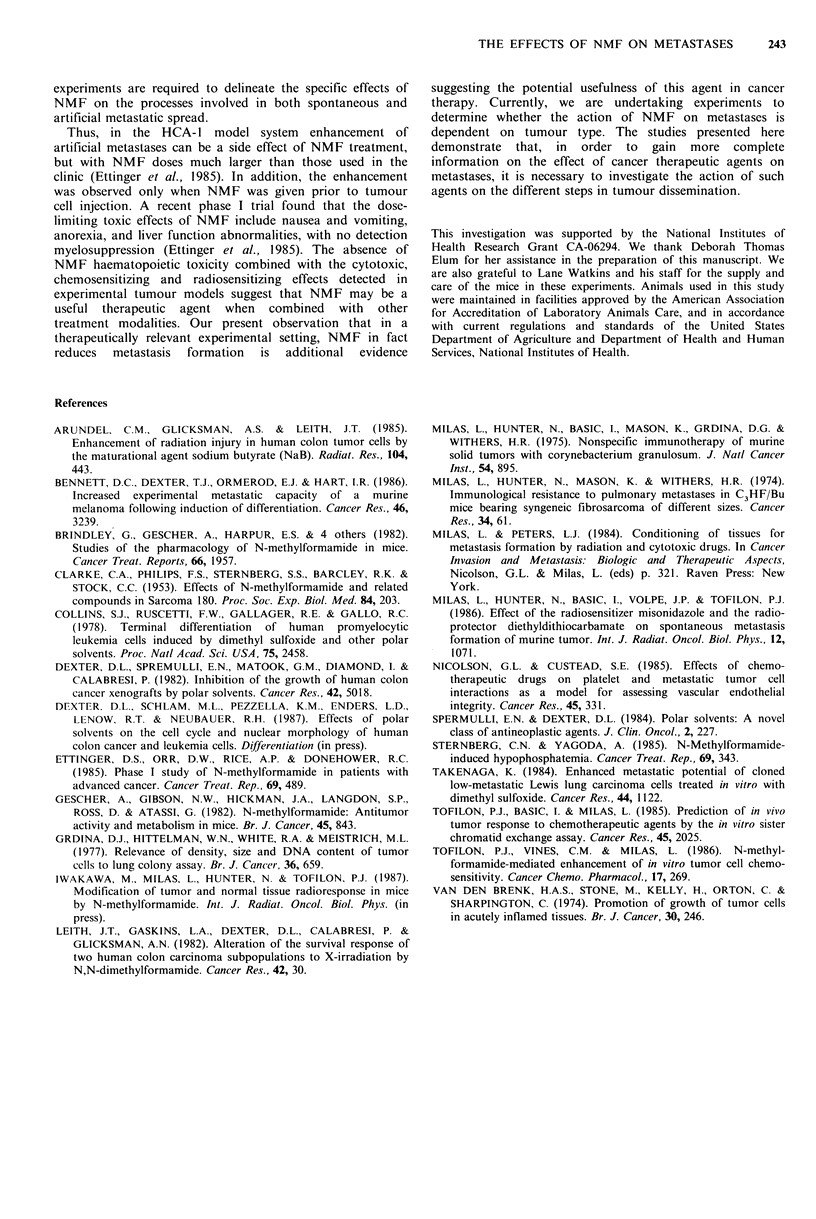# The effects of N-methylformamide on artificial and spontaneous metastases from a murine hepatocarcinoma.

**DOI:** 10.1038/bjc.1987.46

**Published:** 1987-03

**Authors:** P. J. Tofilon, C. M. Vines, L. Milas

## Abstract

The effects of the differentiation-inducing polar solvent N-methylformamide (NMF) on artificially induced and spontaneous metastases from a murine hepatocarcinoma (HCA-1) in C3Hf/Kam mice were investigated. Exposure of HCA-1 cells in vitro for 6 days to 1.0% or 1.25% NMF resulted in an increase in the number of lung nodules formed in mice when these cells were injected into their tail veins. This in vitro NMF exposure increased cell volume and induced only a slight amount of cytotoxicity. Administration of NMF to mice 1 day before i.v. tumour cell inoculation resulted in a dose-dependent increase in the number of lung nodules formed, beginning at an NMF dose of 600 mg kg-1. NMF caused a similar magnitude of metastasis enhancement in immunosuppressed mice. However, when the maximum dose tested (1,800 mg kg-1) was administered as 6 daily fractions of 300 mg kg-1 each, no increase in artificial metastases was detected. Administration of NMF to mice one day after i.v. tumour cell injection resulted in a dose-dependent decrease in the number of lung nodules. In mice bearing 5-6 mm HCA-1 leg tumours, treatment with 6 daily fractions of NMF (300 mg kg-1 each) significantly reduced the number of spontaneous pulmonary metastases, yet had very little effect on the growth of the primary tumour. These data suggest that, in a clinically relevant treatment setting, NMF can reduce metastasis formation.


Br. J. Cancer (1987) 55, 239-243 © The Macmillan Press Ltd., 1987

### The effects of N-methylformamide on artificial and spontaneous metastases from a murine hepatocarcinoma

P.J. Tofilon, C.M. Vines & L. Milas

*Department of Experimental Radiotherapy, Box 66, The University of Texas, M.D. Anderson Hospital and Tumor Institute at Houston, Houston, TX 77030, USA.*

**Summary** The effects of the differentiation-inducing polar solvent N-methylformamide (NMF) on artificially induced and spontaneous metastases from a murine hepatocarcinoma (HCA-1) in $C_3Hf$/Kam mice were investigated. Exposure of HCA-1 cells *in vitro* for 6 days to 1.0% or 1.25% NMF resulted in an increase in the number of lung nodules formed in mice when these cells were injected into their tail veins. This *in vitro* NMF exposure increased cell volume and induced only a slight amount of cytotoxicity. Administration of NMF to mice 1 day before i.v. tumour cell inoculation resulted in a dose-dependent increase in the number of lung nodules formed, beginning at an NMF dose of $600 mg kg^{-1}$. NMF caused a similar magnitude of metastasis enhancement in immunosuppressed mice. However, when the maximum dose tested ($1,800 mg kg^{-1}$) was administered as 6 daily fractions of $300 mg kg^{-1}$ each, no increase in artificial metastases was detected. Administration of NMF to mice one day after i.v. tumour cell injection resulted in a dose-dependent decrease in the number of lung nodules. In mice bearing 5–6 mm HCA-1 leg tumours, treatment with 6 daily fractions of NMF ($300 mg kg^{-1}$ each) significantly reduced the number of spontaneous pulmonary metastases, yet had very little effect on the growth of the primary tumour. These data suggest that, in a clinically relevant treatment setting, NMF can reduce metastasis formation.

Maturational agents are a class of antitumour compounds characterized by their ability to induce malignant cells to form better-differentiated phenotypes. In addition, some of these agents render tumour cells more susceptible to the cytotoxic effects of ionizing radiation (Leith *et al.*, 1982; Iwakawa *et al.*, 1986) and certain antineoplastic drugs (Spremulli & Dexter, 1984; Tofilon *et al.*, 1986). The polar solvent N-methylformamide (NMF) belongs to this class of agents and is currently undergoing phase II trials in the treatment of human tumours (Sternberg & Yagoda, 1985). Exposure of human and rodent leukaemic cells *in vitro* to NMF was found to result in terminal differentiation (Collins *et al.*, 1978). However, cells from most solid tumours exposed to NMF do not terminally differentiate, but rather proceed to form so-called better-differentiated or less malignant phenotypes (Spremulli & Dexter, 1984). Changes in cellular characteristics reported to be associated with the better-differentiated phenotypes include alterations in cell morphology, reduction in the rate of cell proliferation and clonogenicity, and the production of specialized cell products, characteristics usually associated with differentiated cells. However, upon withdrawal of NMF, tumour cells revert to their original form.

In addition to the observed cellular changes, it was hypothesized that the NMF-mediated induction of a better-differentiated state in malignant tumours should be accompanied by less aggressive behaviour of these tumours, including a reduction in metastatic propensity (Spremulli & Dexter, 1984). While the influence of NMF on the growth of several experimental solid tumours has been studied (Clarke *et al.*, 1953; Gescher *et al.*, 1982; Dexter *et al.*, 1982), little is known about the effect of maturational agents on either the formation or therapy of tumour metastases. Cells treated *in vitro* with the maturational agent DMSO (Takenaga, 1984) or exposed to conditions that lead to maturation (Bennett *et al.*, 1986) were reported to have increased lung colonization ability after i.v. injection, suggesting that maturational agents might promote metastasis formation rather than be antimetastatic. Since NMF has already entered clinical trials, it is important to establish whether it influences metastasis formation. We report here studies designed to investigate the effects of NMF on three aspects of metastases of a murine hepatocarcinoma (HCA-1): lung colonization, established artificial micrometastatic foci and spontaneous metastasis.

Exposure of HCA-1 cells *in vitro* results in an increase in cell doubling time, a reduction in clonogenicity and an increase in cell volume (Tofilon *et al.*, 1986), changes associated with a better differentiated phenotype (Spremulli & Dexter, 1984).

Correspondence: P.J. Tofilon.
Received 12 August 1986 and in revised form 23 October 1986.

### Materials and methods

*Mice*

Inbred male $C_3Hf$/Kam mice bred and maintained in our own specific pathogen-free mouse colony were used. Mice were 9 to 12 weeks old at the beginning of each experiment.

*Tumours*

In these studies, a spontaneously developed hepatic carcinoma (HCA-1) syngeneic to $C_3Hf$/Kam mice was used. The tumour, generously provided by Robert Sedlacek (Massachusetts General Hospital, Boston, MA), is highly metastatic and nonimmunogenic (Milas *et al.*, 1986). Single-cell suspensions were prepared by trypsin digestion of non-necrotic tumour tissue (Milas *et al.*, 1974). Viability of cells was greater than 95% as determined by phase contrast microscopy and trypan blue exclusion.

To generate leg tumours, mice each received an injection of $5 \times 10^5$ viable tumour cells into the right hind thigh. When tumours grew to 5–6 mm in diameter, NMF treatment was initiated. To obtain tumour growth curves, three mutually orthogonal diameters of tumours were measured three times per week with a vernier caliper, and the mean values were calculated. Mice were killed 22 days after the initiation of NMF treatment, their lungs were removed, and the number of lung metastases in each was determined.

To produce tumour micrometastases in the lung, $1 \times 10^5$ viable HCA-1 cells were suspended in 0.50 ml of Hsu's medium (Grand Island Biological Co., Grand Island, NY) and injected into the tail vein of each mouse. Fourteen days after tumour cell injection, mice were killed, and the number of lung nodules in each was determined using the method described earlier (Milas *et al.*, 1975).

*NMF treatment* in vivo

NMF was diluted in Solution A (8.0 g NaCl; 0.4 g KCl;

1.0 g glucose; 0.35 g $NaHCO_3$ $l^{-1}$ water), stored in the dark, and administered as an i.p. or s.c. injection. In addition, NMF was also administered through an osmotic minipump (Alza 2001). Minipumps were implanted s.c. using sodium pentobarbital (Nembutal) anaesthesia and delivered 1 $\mu l\,h^{-1}$ of a 313 mg $ml^{-1}$ solution of NMF, which is equivalent to 300 mg $kg^{-1}$ $day^{-1}$. Pumps were removed under anaesthesia 6 days after implantation.

*Cell culture*

Hepatocarcinoma cells were grown in monolayer culture in Hsu's medium containing 20% foetal calf serum and plating efficiency was determined as described elsewhere (Tofilon *et al.*, 1985). Cells were exposed to NMF in normal growth medium for 6 days. Cell volume was determined using a Coulter Channelyzer and the appropriate standards.

### Results

To determine the effects of *in vitro* NMF exposure on HCA-1 artificial metastasis formation, cells were grown in monolayer culture in medium containing 1.0% or 1.25% NMF for 6 days (at which time they were still in exponential phase), were trypsinized, and a single cell suspension of $10^5$ cells was injected into the tail vein of each mouse. A representative experiment is shown in Table I. Compared with controls, exposure of HCA-1 cells *in vitro* to 1.0% or 1.25% NMF resulted in a significant increase in the number of lung nodules when cells were injected i.v. The concentrations of NMF used in this experiment produced only a slight amount of cytotoxicity as determined by *in vitro* colony formation (plating efficiency), yet in the groups that received 1% or 1.25% NMF cell volumes, determined from the single cell suspensions, were significantly greater than control. In an additional experiment, cells were grown in 1% NMF for 6 days and then in NMF-free medium for 3 days before i.v. injection (data not shown). In this case, the cell volume and the number of lung colonies formed were similar to untreated values indicating that, as for other NMF-induced cellular changes (Spremulli & Dexter, 1984; Tofilon *et al.*, 1986), the increase in cell volume and lung colonizing ability are also reversible.

In addition to having influences on the tumour cell, chemotherapeutic agents administered *in vivo* can affect the host, resulting in an increase in metastasis formation (Milas & Peters, 1984). Thus, to determine whether NMF, independent of its effects on the tumour cells, enhances metastasis formation through an effect on the host, NMF was administered before tumour cell inoculation. In this experiment, NMF was delivered in single, graded, i.p. doses 1 day before tumour cell inoculation or as 6 daily bolus treatments with the last treatment administered 1 day before tumour cell inoculation. Brindley *et al.* (1982) found that 24 h after CBA mice were injected i.p. with NMF the plasma level declined to an undetectable concentration, according to a gas chromatographic technique. As shown in Table II, single injections of NMF resulted in a dose-dependent increase in the number of artificial metastases formed, whereas a total dose of 1,800 mg $kg^{-1}$ delivered as 6 daily injections of 300 mg $kg^{-1}$ each did not affect the number of lung nodules as compared to controls.

The time of NMF pretreatment that results in the greatest enhancement of metastases was investigated by administering a single, relatively large dose of NMF (1,200 mg $kg^{-1}$) at various times before i.v. injection of tumour cells. Fourteen days after receiving tumour cell injection, mice were killed and the number of lung nodules was determined. The maximum enhancement of artificial metastases was obtained when NMF was given 1 day before tumour cell injection (Figure 1). Essentially no enhancement was detected when NMF was administered 10 days before tumour cell injection, suggesting that whatever host damage is induced by NMF is repaired within 10 days. However, in contrast to the effect exerted by the pretreatment with NMF, the administration of NMF 1 day after tumour cell injection resulted in a significant reduction in the number of artificial metastases.

Because suppression of the immune system is a mechanism by which cytotoxic agents cause enhancement of artificial metastases, a possibility exists that a similar mechanism is also involved in NMF-induced metastasis enhancement. If such a mechanism is involved, then NMF would be less effective in immunosuppressed animals. To test this hypothesis, mice were immunosuppressed by exposing them to 6 Gy of whole body irradiation (WBI) 4 days before injection of tumour cells. NMF (900 mg $kg^{-1}$) was given on days 4 and 3 (a total dose of 1,800 mg $kg^{-1}$) before tumour cell injection. The results, presented in Table III, show that NMF increased the number of lung nodules by ~2-fold in both unirradiated mice and those given WBI. WBI alone increased the number of lung nodules. Thus, these data suggest that the NMF-induced enhancement of artificial metastases is unlikely to be mediated via suppression of the immune system.

**Table I** Effect of *in vitro* NMF exposure of *in vivo* artificial metastasis formation

| | | In vitro *properties* | |
|---|---|---|---|
| *Treatment* | *Number of lung nodules*[a] | *Plating efficiency*[b] | *Cell volume* ($\mu m^3$) |
| Experiment 1 | | | |
| None | 13.3 ± 3.6 | 0.76 ± 0.05 | 949 |
| NMF (1%) | 209.0 ± 83.3[c] | 0.69 ± 0.03 | 1548 |
| NMF (1.25%) | 184.6 ± 35.5[c] | 0.66 ± 0.03 | 1475 |

HCA-1 cells were grown *in vitro* in normal growth medium or medium containing NMF (1% or 1.25%) for 6 days. Cells were then trypsinized; $1 \times 10^5$ cells were suspended in 0.5 ml Hsu's medium and injected into the tail veins of mice. Fourteen days after tumour cell injection the mice were killed and the number of lung nodules was determined. The *in vitro* plating efficiency and cell volume were also determined for each treatment group, using the same cell suspensions. Therefore, in the case of cell volume, only one suspension was used. It should be noted that the effect of NMF on cell volume is consistently reproducible (Tofilon *et al.*, 1986).

[a]Values represent the mean ± s.e. The control group and the group that received 1.25% NMF contained 7 mice each, whereas the group that received 1% NMF contained 3 mice. [b]Values represent the mean of ± s.e. for 4 petri dishes. [c]Significantly different from untreated as determined by Student's $t$ test ($P < 0.001$).

**Table II** Effects of pretreatment on artificial metastases

| *NMF pretreatment* ($mg\,kg)^{-1}$ | *Number of lung nodules* |
|---|---|
| Untreated | 21.3 ± 2.1 |
| 150 | 18.2 ± 2.9 |
| 300 | 21.9 ± 2.9 |
| 600 | 33.5 ± 2.5[a] |
| 1200 | 84.9 ± 16.0[a] |
| 6 × 300 | 21.7 ± 4.7 |

An HCA-1 leg tumour was excised, a single cell suspension was generated, and $1 \times 10^5$ cells were injected into the tail veins of the mice. Fourteen days later the mice were killed and the number of lung nodules was determined. NMF was administered as a single i.p. injection 1 day before, or with the last dose of the 6-daily-dose protocol given 1 day before, tumour cell injection. Values represent the mean ± s.e. for 6 to 7 mice.

[a]Significantly different from untreated as determined by Student's $t$ test ($P < 0.01$).

**Figure 1** HCA-1 cells ($10^5$) obtained from an *in vivo* tumour cell suspension, were injected into the tail veins of mice; 14 days later the mice were killed and the number of lung nodules was determined. NMF (1,200 mg kg$^{-1}$) was administered in a single i.p. injection at various times before tumour cell injection (day 0) and 1 day after tumour cell injection (closed symbols). The open symbol represents results from untreated mice. Values are the mean ±s.e. for 6 to 8 mice.

**Table III** Effect of NMF pretreatment on artificial metastasis formation in whole body irradiation (WBI) mice

| Pretreatment | Number of lung $10^5$ cells | $10^4$ cells |
|---|---|---|
| Untreated | 81.9± 3.7 | 7.7±1.0 |
| NMF (900 mg kg$^{-1}$) | 130.9±22.3 | 13.5±1.8 |
| WBI (6 Gy) | 155.3±21.7 | 17.6±3.1 |
| WBI+NMF | 314.3±51.1 | 35.5±6.4 |

HCA-1 cells ($10^4$ or $10^5$), obtained from an *in vivo* tumour cell suspension, were injected into the tail veins of mice; 14 days later the mice were killed and the number of lung nodules was determined. WBI (6 Gy) was administered 4 days before tumour cell injection, and NMF (900 mg kg$^{-1}$) was given on days 4 and 3 before tumour cell injection. Values represent the mean ±s.e. for 7 to 8 mice.

The data shown in the Figure 1 indicate that, whereas NMF pretreatment increased metastases formation, administration of NMF 1 day after tumour cell injection resulted in a decrease in the number of lung nodules. To further investigate the effects of NMF on existing artificial metastases, 1 day after tumour cell injection, NMF was administered as a single i.p. injection or daily treatment was initiated. Mice were killed 14 days after tumour cell injection, at which time the lung nodules in all groups were approximately the same size. Administration of single injections of NMF resulted in a dose-dependent decrease in the number of lung nodules (Figure 2). Administration of a total NMF dose of 1,800 mg kg$^{-1}$ divided into 2, 3, or 6 daily fractions also reduced the number of lung nodules; however, the reduction was not as great as with that same total dose delivered in a single injection.

Data from artificial metastases experiments indicate that NMF delivered in 6 daily doses of 300 mg kg$^{-1}$ each *before* tumour cell injection does not result in the enhancement of metastases, but if given *after* injection, reduces the number

**Figure 2** HCA-1 cells ($10^5$), obtained from *in vivo* tumour cell suspension, were injected into the tail vein of mice; 14 days later the mice were killed and the number of lung nodules determined. NMF was administered in graded doses (i.p.) 1 day after tumour cell injection (closed symbols); daily injections (2×900 mg kg$^{-1}$, 3×600 mg kg$^{-1}$ or 6×300 mg kg$^{-1}$) were begun 1 day after tumour cell injection (open symbols). Values represent the mean ±s.e. for 6 to 7 mice.

**Table IV** Effects of NMF on spontaneous metastases

| NMF treatment (mg kg$^{-1}$) | Number of lung nodules | Tumour diameter (mm)[a] |
|---|---|---|
| Untreated | 12.8±2.4 | 19.6±0.7 |
| 6×300, i.p. | 4.3±0.8[b] | 18.4±0.6 |
| 6×300, s.c. | 6.5±1.2[b] | 19.3±1.2 |
| 6×300, pump | 6.3±1.2[b] | 18.5±1.8 |

HCA-1 leg tumours were generated by injecting $5\times10^5$ cells (obtained from an *in vivo* tumour cell suspension) into the hind right leg of each mouse. When tumours were 5–6 mm in diameter NMF treatment was initiated and continued for 6 daily i.p. or s.c. injections of 300 mg kg$^{-1}$ each or through continual release from an osmotic minipump implanted s.c. that delivered 300 mg kg$^{-1}$ day$^{-1}$ for 6 days. Values represent the mean ±s.e. for 8 to 9 mice. Mice were killed 22 days after the initiation of NMF treatment, and the number of lung nodules was determined.

[a]Mean tumour diameter at the time mice were killed. [b]Significantly different from control as determined by Student's *t* test ($P<0.01$).

of lung nodules. Thus, this treatment schedule was used to determine the effects of NMF on spontaneous pulmonary metastases from HCA-1 tumours in the leg. In this experiment, to investigate the effects of the route of administration, NMF treatment was initiated when tumours were 5–6 mm in diameter and, consisted of 6 daily i.p. or s.c. injections of 300 mg kg$^{-1}$ or 300 mg kg$^{-1}$ day$^{-1}$ delivered for 6 days by an s.c. implanted minipump. Mice were killed 22 days after the initiation of NMF treatment, and the number of lung nodules was determined. For each route of NMF delivery, 300 mg kg$^{-1}$ × 6 days resulted in a significant reduction in the number of spontaneous metastases compared with that for untreated mice (Table IV). However, there was no differences in the sizes of the metastases between control and treatment groups (data not shown). NMF treatment slowed tumour growth only slightly, with all tumours being approximately the same size at the time the mice were killed. These data suggest that NMF has anti-metastatic actions, yet has very little detectable effect on the growth of the primary tumour.

In an additional experiment, mice bearing HCA-1 leg tumours 5–6 mm in diameter were given 6 daily i.p. injections of NMF ($300\,mg\,kg^{-1}$ each); on the seventh day mice were killed, a tumour cell suspension was prepared, and the average cell volume in the suspension was determined according to a Coulter Channelyzer. The tumour cell suspension generated from untreated mice had an average cell volume of $480\,\mu m^3$, whereas that generated from mice that had received NMF treatment had an average cell volume of $1{,}700\,\mu m^3$. It appears that, as in the *in vitro* experiments in Table I, NMF treatment *in vivo* also results in an increase in tumour cell volume.

### Discussion

Depending on the experimental setting, NMF exerted both enhancing and inhibiting effects on tumour metastasis. The enhancement was observed when mice were treated with NMF before i.v. inoculation of tumour cells, whereas reduction resulted when the drug was given after tumour cell inoculation. This pattern of metastatic effects is very similar to that reported for a number of cytotoxic agents (Milas & Peters, 1984). These authors discussed in detail a number of host mediated possibilities responsible for the enhancement of metastasis formation caused by cytotoxic agents, which included immunosuppression, stress-like reaction, and local capillary damage. Immunosuppression as a possible mechanism for NMF induced metastasis enhancement can be excluded on the basis that the HCA-1 tumour is not immunogenic (Milas *et al.*, 1986), and that it caused a similar magnitude of metastasis enhancement in both normal and WBI-immunosuppressed mice (Table III). Although the tumour is not immunogenic, its cells generated more colonies in WBI than in normal mice, which can be attributed largely to pulmonary vasculature damage (Milas & Peters, 1984). Although local tissue damage and host mediated mechanisms, other than immunosuppression, may be responsible for the NMF-induced enhancement of metastases, it is also quite possible that, analogous to many other iatrogenic agents that enhance metastasis formation, NMF acted through the damage of endothelial cells of the lung vasculature (Milas *et al.*, 1984; Nicolson & Custead, 1985) and (or) through exerting a stress-like reaction upon the host (Van den Brenk *et al.*, 1974).

A direct effect on tumour cells is also a potential mechanism by which NMF increased metastasis formation. Exposure of HCA-1 cells *in vitro* to noncytotoxic concentrations of NMF followed by injection of the cells into the tail veins of mice resulted in an increase in the number of lung nodules formed compared with that when untreated cells were used. In the study by Takenaga using Lewis lung cells (Takenaga, 1984), the increase in artificial metastases was attributed to a DMSO-mediated increase in cell adhesiveness and degradative enzyme activity. We did not measure enzyme activity or cell adhesiveness in HCA-1 cells; however, cell volume was determined and found to be greater for cells exposed to NMF. The exposure of a colon adenocarcinoma cell line to NMF was also found to result in an increase in cell volume (Arundel *et al.*, 1986). Since tumour cell volume is a factor influencing tumour cell arrest in the pulmonary vasculature, with larger cells having a higher probability for arrest and subsequent metastasis formation (Grdina *et al.*, 1977), it is reasonable to assume that NMF may have increased the formation of artificial metastases in the present study partly due to the increase in cell volume. It is not known why NMF increases cell volume. The possibility that it acts through accumulation of cells in a specific phase of the cell cycle can be excluded since NMF does not perturb cell cycle distribution of either HCA-1 cells (unpublished observation) or other tumour cells (Dexter *et al.*, 1987). Whatever mechanism is involved, the effect of NMF on the ability of cells to exhibit enhanced lung colonization was transient and was lost by day 3 after the removal of NMF.

As already mentioned, the dose of NMF is an important variable in the induction of metastases enhancement: doses smaller than $600\,mg\,kg^{-1}$ are not effective even if given in multiple injections. However, whereas NMF administered in 6 daily doses of $300\,mg\,kg^{-1}$ each before tumour cell injection did not enhance metastases formation, this NMF treatment protocol administered 1 day after tumour cell injection did result in a significant reduction in the number of lung nodules. In the absence of the production of specific cell products indicating differentiation, it is not possible based on this experiment to determine whether the reduction in artificial metastases was the result of the induction of a better-differentiated phenotype or of direct cytotoxicity. However, when NMF ($6 \times 300\,mg\,kg^{-1}$) was administered to mice bearing HCA-1 leg tumours of 5–6 mm in diameter, only a slight growth delay was detected. The difference in NMF effects on artificial metastases and solid tumours may be the result of a difference in tumour cell load. Gescher *et al.* (1982) found significant antitumour activity against certain murine tumours when NMF treatment was initiated 1 day after i.m. tumour cell injection, before tumours were palpable. Thus, similar to our findings in the artificial metastases experiment, these authors found NMF to be effective against a relatively small tumour cell load.

As for the *in vitro* exposure to NMF, the average cell volume in a tumour cell suspension obtained from NMF-treated mice was also increased compared with that from controls. As was discussed for artificial metastases, the increase in tumour cell size might be expected to result in an increase in spontaneous metastases. However, cell volume was determined in a tumour suspension; whether the volume of the tumour cells in the actual *in situ* tumour is increased remains to be determined. With respect to the influence of cell size on metastasis formation, it was expected that the size of the tumour cells shed into the circulation would be of major importance rather than the size of cell remaining in the primary tumour.

NMF treatment of mice bearing HCA-1 leg tumours had only a slight effect on the growth of the primary tumour, yet significantly reduced the number of spontaneous metastases. Although it is not possible to conclusively eliminate a cytotoxic action on the initial lung metastases formed during the 6-day NMF treatment period, it is tempting to speculate that the reduction in spontaneous metastases was due to a direct effect on the primary tumour. It is possible that NMF treatment results in a decrease in the release of tumour cells into the circulation. While NMF caused only a slight reduction in the size of leg tumours, it increased cell volume by $\sim 1.6$ times. Thus, NMF-treated tumours are likely to contain significantly fewer cells than untreated tumours of the same size; consequently, the probability of the release of cells into the circulation is less in the treated tumours. In addition, if NMF increases the adhesiveness of tumour cells, as DMSO does (Takenaga, 1984), then the likelihood for their release from the primary tumour is also minimized. As previously postulated (Spremulli & Dexter, 1984), NMF may induce tumour cells to form a better-differentiated phenotype and, thus, to be less aggressive, resulting in a decrease in metastatic potential. *In vitro*, the induction of a better-differentiated phenotype by NMF is accompanied by an increase in cell volume (Arundel *et al.*, 1985; Tofilon *et al.*, 1986): the detected increase in cell volume *in vivo* may reflect a similar change in phenotype. Obviously, further experiments are required to delineate the specific effects of NMF on the processes involved in both spontaneous and artificial metastatic spread.

Thus, in the HCA-1 model system enhancement of artificial metastases can be a side effect of NMF treatment, but with NMF doses much larger than those used in the clinic (Ettinger *et al.,* 1985). In addition, the enhancement was observed only when NMF was given prior to tumour cell injection. A recent phase I trial found that the dose-limiting toxic effects of NMF include nausea and vomiting, anorexia, and liver function abnormalities, with no detection myelosuppression (Ettinger *et al.,* 1985). The absence of NMF haematopoietic toxicity combined with the cytotoxic, chemosensitizing and radiosensitizing effects detected in experimental tumour models suggest that NMF may be a useful therapeutic agent when combined with other treatment modalities. Our present observation that in a therapeutically relevant experimental setting, NMF in fact reduces metastasis formation is additional evidence suggesting the potential usefulness of this agent in cancer therapy. Currently, we are undertaking experiments to determine whether the action of NMF on metastases is dependent on tumour type. The studies presented here demonstrate that, in order to gain more complete information on the effect of cancer therapeutic agents on metastases, it is necessary to investigate the action of such agents on the different steps in tumour dissemination.

This investigation was supported by the National Institutes of Health Research Grant CA-06294. We thank Deborah Thomas Elum for her assistance in the preparation of this manuscript. We are also grateful to Lane Watkins and his staff for the supply and care of the mice in these experiments. Animals used in this study were maintained in facilities approved by the American Association for Accreditation of Laboratory Animals Care, and in accordance with current regulations and standards of the United States Department of Agriculture and Department of Health and Human Services, National Institutes of Health.

### References

ARUNDEL, C.M., GLICKSMAN, A.S. & LEITH, J.T. (1985). Enhancement of radiation injury in human colon tumor cells by the maturational agent sodium butyrate (NaB). *Radiat. Res.*, **104,** 443.

BENNETT, D.C., DEXTER, T.J., ORMEROD, E.J. & HART, I.R. (1986). Increased experimental metastatic capacity of a murine melanoma following induction of differentiation. *Cancer Res.*, **46,** 3239.

BRINDLEY, G., GESCHER, A., HARPUR, E.S. & 4 others (1982). Studies of the pharmacology of N-methylformamide in mice. *Cancer Treat. Reports,* **66,** 1957.

CLARKE, C.A., PHILIPS, F.S., STERNBERG, S.S., BARCLEY, R.K. & STOCK, C.C. (1953). Effects of N-methylformamide and related compounds in Sarcoma 180. *Proc. Soc. Exp. Biol. Med.* **84,** 203.

COLLINS, S.J., RUSCETTI, F.W., GALLAGER, R.E. & GALLO, R.C. (1978). Terminal differentiation of human promyelocytic leukemia cells induced by dimethyl sulfoxide and other polar solvents. *Proc. Natl Acad. Sci. USA,* **75,** 2458.

DEXTER, D.L., SPREMULLI, E.N., MATOOK, G.M., DIAMOND, I. & CALABRESI, P. (1982). Inhibition of the growth of human colon cancer xenografts by polar solvents. *Cancer Res.*, **42,** 5018.

DEXTER, D.L., SCHLAM, M.L., PEZZELLA, K.M., ENDERS, L.D., LENOW, R.T. & NEUBAUER, R.H. (1987). Effects of polar solvents on the cell cycle and nuclear morphology of human colon cancer and leukemia cells. *Differentiation* (in press).

ETTINGER, D.S., ORR, D.W., RICE, A.P. & DONEHOWER, R.C. (1985). Phase I study of N-methylformamide in patients with advanced cancer. *Cancer Treat. Rep.*, **69,** 489.

GESCHER, A., GIBSON, N.W., HICKMAN, J.A., LANGDON, S.P., ROSS, D. & ATASSI, G. (1982). N-methylformamide: Antitumor activity and metabolism in mice. *Br. J. Cancer,* **45,** 843.

GRDINA, D.J., HITTELMAN, W.N., WHITE, R.A. & MEISTRICH, M.L. (1977). Relevance of density, size and DNA content of tumor cells to lung colony assay. *Br. J. Cancer,* **36,** 659.

IWAKAWA, M., MILAS, L., HUNTER, N. & TOFILON, P.J. (1987). Modification of tumor and normal tissue radioresponse in mice by N-methylformamide. *Int. J. Radiat. Oncol. Biol. Phys.* (in press).

LEITH, J.T., GASKINS, L.A., DEXTER, D.L., CALABRESI, P. & GLICKSMAN, A.N. (1982). Alteration of the survival response of two human colon carcinoma subpopulations to X-irradiation by N,N-dimethylformamide. *Cancer Res.*, **42,** 30.

MILAS, L., HUNTER, N., BASIC, I., MASON, K., GRDINA, D.G. & WITHERS, H.R. (1975). Nonspecific immunotherapy of murine solid tumors with corynebacterium granulosum. *J. Natl Cancer Inst.*, **54,** 895.

MILAS, L., HUNTER, N., MASON, K. & WITHERS, H.R. (1974). Immunological resistance to pulmonary metastases in $C_3$HF/Bu mice bearing syngeneic fibrosarcoma of different sizes. *Cancer Res.*, **34,** 61.

MILAS, L. & PETERS, L.J. (1984). Conditioning of tissues for metastasis formation by radiation and cytotoxic drugs. In *Cancer Invasion and Metastasis: Biologic and Therapeutic Aspects,* Nicolson, G.L. & Milas, L. (eds) p. 321. Raven Press: New York.

MILAS, L., HUNTER, N., BASIC, I., VOLPE, J.P. & TOFILON, P.J. (1986). Effect of the radiosensitizer misonidazole and the radio-protector diethyldithiocarbamate on spontaneous metastasis formation of murine tumor. *Int. J. Radiat. Oncol. Biol. Phys.*, **12,** 1071.

NICOLSON, G.L. & CUSTEAD, S.E. (1985). Effects of chemo-therapeutic drugs on platelet and metastatic tumor cell interactions as a model for assessing vascular endothelial integrity. *Cancer Res.*, **45,** 331.

SPERMULLI, E.N. & DEXTER, D.L. (1984). Polar solvents: A novel class of antineoplastic agents. *J. Clin. Oncol.*, **2,** 227.

STERNBERG, C.N. & YAGODA, A. (1985). N-Methylformamide-induced hypophosphatemia. *Cancer Treat. Rep.*, **69,** 343.

TAKENAGA, K. (1984). Enhanced metastatic potential of cloned low-metastatic Lewis lung carcinoma cells treated *in vitro* with dimethyl sulfoxide. *Cancer Res.*, **44,** 1122.

TOFILON, P.J., BASIC, I. & MILAS, L. (1985). Prediction of *in vivo* tumor response to chemotherapeutic agents by the *in vitro* sister chromatid exchange assay. *Cancer Res.*, **45,** 2025.

TOFILON, P.J., VINES, C.M. & MILAS, L. (1986). N-methyl-formamide-mediated enhancement of *in vitro* tumor cell chemo-sensitivity. *Cancer Chemo. Pharmacol.*, **17,** 269.

VAN DEN BRENK, H.A.S., STONE, M., KELLY, H., ORTON, C. & SHARPINGTON, C. (1974). Promotion of growth of tumor cells in acutely inflamed tissues. *Br. J. Cancer,* **30,** 246.